# Molecular Characteristics of *Staphylococcus aureus* Causing Bovine Mastitis between 2014 and 2015

**DOI:** 10.3389/fcimb.2017.00127

**Published:** 2017-04-19

**Authors:** Tianming Li, Huiying Lu, Xing Wang, Qianqian Gao, Yingxin Dai, Jun Shang, Min Li

**Affiliations:** ^1^Department of Laboratory Medicine, Renji Hospital, School of Medicine, Shanghai Jiaotong UniversityShanghai, China; ^2^Department of Laboratory Medicine, Shanghai Children's Medical Center, Shanghai Jiaotong University School of MedicineShanghai, China; ^3^Shanghai Institute for Veterinary Drug and Feeds ControlShanghai, China

**Keywords:** bovine mastitis, *S. aureus*, MLST-genotyping, SCCmec typing, antimicrobial resistance

## Abstract

*Staphylococcus aureus* is highly pathogenic and can cause diseases in both humans and domestic animals. In animal species, including ruminants, *S. aureus* may cause severe or sub-clinical mastitis. This study aimed to investigate the molecular profile, antimicrobial resistance, and genotype/phenotype correlation of 212 *S. aureus* isolates recovered from cases of bovine mastitis from 2014 to 2015 in the Shanghai and Zhejiang areas of China. Nineteen sequence types (STs) were determined by multi-locus sequence typing, while the dominant ST was ST97, followed by ST520, ST188, ST398, ST7, and ST9. Within 14 methicillin-resistant *S. aureus* (MRSA) isolates and 198 methicillin-susceptible *S. aureus* (MSSA) isolates, ST97 was the predominant MSSA clone and ST9-MRSA-SCCmecXII-spa t899 was the most common MRSA clone. The MRSA strains showed much higher rates of resistance to multiple antibiotics than did MSSA strains. Compared with other MSSA strains, MSSA ST398 was more resistant to clindamycin, erythromycin, and ciprofloxacin. No isolates were resistant to vancomycin, teicoplanin, or linezolid. The molecular profiles of the virulence genes varied in different strains. ST520 strains carried seg-sei-sem-sen-seo genes, and ST9 and ST97 harbored sdrD-sdrE genes. Virulence phenotype analysis showed diversity in different clones. Biofilm formation ability was significantly enhanced in ST188 and ST7, and red blood cell lysis capacity was relatively strong in all *S. aureus* strains of animal origin except ST7. Our results indicate that MSSA was the predominant *S. aureus* strain causing bovine mastitis in eastern regions of China. However, the presence of multidrug resistant and toxigenic MRSA clone ST9 suggests that comprehensive surveillance of *S. aureus* infection should be implemented in the management of animal husbandry products.

## Introduction

*Staphylococcus aureus* is a common facultative pathogenic bacterium that has long been recognized as a challenge in both human and veterinary medicine (Nemeghaire et al., 2014). In cattle, it is responsible for approximately one-third of cases of clinical and subclinical mastitis (Bradley et al., 2007; Botrel et al., 2010), a disease that causes major economic loss in the dairy industry worldwide. Since MRSA was first reported in the United Kingdom in 1961, it has become a global cause of hospital-associated (HA) and community-associated (CA) infections (Uhlemann et al., 2014). In the past few decades, a new subgroup of *S. aureus*, so-called livestock-associated *S. aureus* (LA-SA) has been described. There have been several reports of MRSA colonization and/or infections in dairy cattle since the first report of MRSA in mastitis in 1972 (Devriese et al., 1972; Monecke et al., 2007; Fessler et al., 2010; Huber et al., 2010). LA-SA ST398 was originally reported to have emerged in the Netherlands among pigs and pig farmers in 2003 (Voss et al., 2005), and was later found in Austria, Germany, and Denmark (Van Cleef et al., 2011; Bal et al., 2016). It developed into an overwhelmingly dominant lineage in Europe and North America (Vanderhaeghen et al., 2010; Cuny et al., 2013). Unlike those in Europe and North America, the epidemic LA MRSA clones in Asian countries have their own characteristics. LA MRSA ST9 has been predominantly isolated from pigs in China, Hong Kong, Taiwan, Thailand, and Malaysia. LA MRSA ST221 has been reported in Japan, and LA MRSA ST398 has emerged among pigs in Singapore and South Korea (Cui et al., 2009; Guardabassi et al., 2009; Neela et al., 2009; Baba et al., 2010; Ho J. et al., 2012; Larsen et al., 2012). Although the sequence type of LA MRSA isolates was mainly ST9 in most Asian countries, these isolates harbored different SCC*mec* elements in different areas, such as SCC*mec* III in China, SCC*mec* IVb, or V in Hong Kong, SCC*mec* V in Malaysia, SCC*mec* IX in Thailand and non-types I–VII in Taiwan (Larsen et al., 2012; Fang et al., 2014; Chuang and Huang, 2015). ST9 strains were usually associated with the MDR phenotype, with high resistance rates (>80%) to erythromycin, clindamycin, tetracycline, ciprofloxacin and gentamicin (Wan et al., 2013). To date, most studies on LA *S. aureus* have been performed in pigs, mainly with MRSA strains, even though the vast majority of *S. aureus* strains are host-specific. The data on molecular typing and antibiotic-related studies of bovine derived *S. aureus* are very limited. A relatively higher incidence of *S. aureus* mastitis has been reported in China than in other countries, which may cause a particular public threat (Li et al., 2011; He et al., 2014). According to previous studies performed in six Chinese provinces from March 2010 to August 2013, the most popular *S. aureus* clones causing bovine mastitis were primarily ST97 (51.9%), ST398 (13.6%) and ST2154 (8.6%), with ST97-MRSA-SCC*mec* IV representing the most common clone (50%; Wang et al., 2015). However, the characteristics of *S. aureus* from dairy cows in eastern regions of China have not yet been thoroughly discussed. To provide elementary evidence for developing the appropriate treatment and control measures for bovine mastitis, it is essential to understand the molecular epidemiology and the antibiotic resistance of *S. aureus* infections locally. Therefore, the present study was designed to analyze the molecular characteristics of *S. aureus* strains in bovine mastitis isolated from 2014 to 2015 in the Shanghai and Zhejiang areas.

## Materials and methods

### Sample collection and bacterial isolation

In total, 212 *S. aureus* isolates were isolated from dairy cows with mastitis from 2014 to 2015 in farms in the Shanghai and Zhejiang areas of China. Milk samples were taken from cows with clinical mastitis that had symptoms including color change in the milk, inflammation of the udder, and decreased milk production. For milk collection, the udders of the clinical mastitis cows were cleaned with water and dried. Cotton balls with 70% ethanol were used to disinfect the surfaces of the udder. The first few streams of milk were dropped. The collected milk was kept in a cooler with ice and transported to the laboratory within 8 h. The milk samples were cultured on 5% blood plate and inoculated at 37°C for 24 h. *S. aureus* identification was based on Gram staining, classical microbiological tests, including catalase and coagulase activity, and were further characterized using VITEK 2 Compact GP ID Card (bioMérieux, Marcy l'Etoile, France). *S. aureus* ATCC43300 was used as a quality control organism. All *S. aureus* isolates were stored at −80°C.

### DNA extraction, MRSA identification, *spa* typing, and SCC*mec* typing

DNA was extracted as previously described (Hartmann et al., 1997). MRSA identification and *mecA* gene detection were performed using a previously published triplex PCR (Maes et al., 2002). The strains were further characterized by *spa* typing, as previously described (Harmsen et al., 2003). The resulting *spa* type was assigned by submitting the data to the *S. aureus spa* type database (http://www.spaserver.ridom.de). Staphylococcal cassette chromosome *mec* (SCC*mec*) types were determined by means of two multiplex PCRs (M-PCRs) designed for the detection of the *mec*-complex and the *ccr*-complex (Kondo et al., 2007; Yan et al., 2016). Specific primers, as previously described (Wu et al., 2015) was used in isolates with SCC*mec* types that could not be determined using the SCC*mec* typing strategy as previously described (Kondo et al., 2007).

### MLST typing and goeBURST algorithm

Isolates were screened using a previously described method (Enright and Spratt, 1999) to detect the following seven housekeeping genes: carbamate kinase (*arcC*), shikimate dehydrogenase (*aroE*), glycerol kinase (*glp*), guanylate kinase (*gmk*), phosphate acetyltransferase (*pta*), triosephosphate isomerase (*tpi*), and acetyl coenzyme A acetyltransferase (*yqiL*). The sequences of the PCR products were compared with the existing sequences available from the MLST website (http://www.mlst.net) for *S. aureus*, and the allelic number was determined for each sequence. The goeBURST algorithm (http://goeBURST.phyloviz.net) was used to infer the evolutionary relatedness of the STs.

### PFGE typing

PFGE was used to compare the genetic diversity of the 10 isolates of ST9 (one isolate of MSSA ST9 and nine isolates of MRSA ST9). Briefly, *Sma* I-digested DNA embedded in agarose plugs was subjected to PFGE analysis at 14°C in a CHEF-MAPPER system (Bio-Rad) at 6 V/cm, in 0.5 × Tris-borate-EDTA buffer, in two stages: first stage, initial pulse, 5 s; final pulse, 15 s for 10 h; second stage, initial pulse, 15 s, final pulse, 60 s for 10 h; angle 120°.

### Antimicrobial susceptibility testing

The standard disk diffusion method was used to test the antibiotic susceptibility of all isolates, and the results were interpreted in accordance with the Clinical and Laboratory Standards Institute (CLSI) guidelines (Ceriotti et al., 2012). Antibiogram classifications were made on the basis of susceptibility to 13 antimicrobials: gentamycin (CN), cefazolin (KZ), cefuroximesodium (CXM), oxacillin (OX), sulfamethoxazole + trimethoprim (SXT), penicillin (P), clindamycin (DA), erythromycin (E), cefoxitin (FOX), ciprofloxacin (CIP), teicoplanin (TEC), vancomycin (VA), and linezolid (LZD).

### Detection of virulence genes

PCR amplification for virulence genes was performed in the dominant STs (the number was >10) for the following 30 staphylococcal virulence genes: the staphylococcal enterotoxin genes (*sea, seb, sec, sed, see, seg, seh, sei, sem, sen, seo, seq, sek*), the toxic shock syndrome toxin (*tsst*), the arginine catabolic mobile gene (*arcA*), the exfoliative toxin genes (*eta, etb*), leukotoxin (*lukF/S, lukE, lukM*; Lina and Etienne, 1999), the hemolysin genes (*hla, hlb, hld, hlg*), the serine protease (*sspA*) and the adhesion genes (*clfA, icaA, fnbA, sdrC*, and *sdrE*) as previously described (Arvidson and Tegmark, 2001; Peacock et al., 2002; Wardenburg et al., 2007). The amplification was carried out on a GeneAmp 9700 thermal cycler (Applied Biosystems, NY, USA) under the following conditions: an initial 5 min denaturation at 94°C, followed by 35 cycles of 30 s at 94°C, 30 s at 55°C, and 30 s at 72°C, with a final extension at 72°C for 7 min. In each PCR, a positive control and a negative control (distilled water) were included. The PCR fragments were visualized by agarose gel electrophoresis and ethidium bromide staining.

### Semiquantitative biofilm assay

A semiquantitative biofilm assay was performed using 96-well tissue culture plates based on the method described by Wang et al. (Wang and Gao, 2008) with the following modification. After the cells were fixed in Bouin's fixative for 1 h, the cells were washed gently two times in phosphate-buffered saline and then stained with 0.1% crystal violet solution. The stain was washed off gently under slowly running water and the plates were dried at room temperature. The absorbance of the stained biofilm was measured at 560 nm using Synergy2 (Bio-Tek).

### Human erythrocyte lysis assay

Human blood was obtained from healthy people during physical examinations in Renji Hospital, Shanghai Jiao Tong University School of Medicine, Shanghai, China with ethical consent. Supernatants were collected from bacterial cultures grown for 8 h. Hemolytic activities were determined by incubating samples with human erythrocytes (2% v/v in Dulbecco's phosphate buffered saline, PBS) for 1 h at 37°C. Hemolysis was determined by measuring the optical density at 540 nm using an enzyme-linked immunosorbent assay reader.

### Statistical analysis

Statistical analyses were performed using Stata software (version10.1/SE, Stata Corp., College Station, TX, USA). We used the χ^2^ and Fisher's exact tests, as appropriate for the analysis of categorical data. Unpaired two-tailed Student's *t*-tests were performed to analyze the statistical significance of biofilm formation and human erythrocytes lysis in *S. aureus* strains. *P* < 0.05 was considered statistically significant.

## Results

### MLST, *spa*, SCC*mec* types

In total, 212 *S. aureus* isolates from bovine mastitis were collected in this study, including 198 (93.4%) MSSA and 14 (6.6%) MRSA isolates. Information associated with the isolation and identification of MSSA, MRSA, *spa* types, MLST, and SCC*mec* types is shown in Table 1. Fourteen isolates were confirmed as MRSA by the cefoxitin disc diffusion test and as *mecA*-positive by PCR. Nineteen distinct STs were identified within the 212 isolates, with ST97 as the most frequently represented isolate (39.2%, 83/212), accounting for more than one third of all *S. aureus* isolates, followed by ST520 (16%, 34/212), ST188 (10%, 21/212), ST398 (7.1%, 15/212), ST7 (5.2%, 11/212), and ST9 (4.7%, 10/212). The *spa* typing discriminated 212 *S. aureus* isolates into 42 *spa* types. Among these, *spa t*267 was the most common type (18.9%, 40/212), followed by *t9303* (13.2%, 28/212), *t359* (10.8%, 23/212), *t189* (8.0%, 17/212), *t034* (4.7%, 10/212), *t091* (4.7%, 10/212), *t899* (4.7%, 10/212), and *t163* (2.8%, 6/212). Each of the remaining *spa* types had no more than five isolates. By SCC*mec* typing, only three types (types IV, V, and XII) were found among 14 MRSA isolates. Type XII was the most predominant type XII and was found in nine isolates (64.3%, 9/14). Type IV was found in four isolates (28.6%, 4/14), and type V was found in one isolate (7.1%, 1/14).

**Table 1:** **Clonal distribution of *S. aureus* causing bovine mastitis in the Shanghai area of China**.

**CCs**	**STs (*n*, %)**	***Spa* type (*n*, %)**	**MSSA (*n*)**	**MRSA (*n*)**	**SCC*mec* type**
CC97	ST97 (83, 39.2)	t267 (40, 18.9)	40	0	/
		t359 (23, 10.8)	23		
		t2802 (5, 2.4)	5		
		t522 (3, 1.4)	3		
		t521 (2, 0.9)	2		
		t1190 (1, 0.5)	1		
		t1201 (1, 0.5)	1		
		t2734 (1, 0.5)	1		
		t730 (1, 0.5)	1		
		t458 (1, 0.5)	1		
		NT (5, 2.4)	5		
CC520	ST520 (34, 16.0)	t9303 (28, 13.2)	28	0	/
		NT (5, 2.4)	5		
		t3104 (1, 0.5)	1		
CC188	ST188 (21, 10.0)	t189 (17, 8.0)	17	0	/
		t803 (3, 1.4)	3		
		t8914 (1, 0.5)	1		
CC398	ST398 (15, 7.1)	t034 (10, 4.7)	10	0	/
		t011 (2, 0.9)	2		
		t1451 (2, 0.9)	2		
		t3041 (1, 0.5)	1		
CC7	ST7 (11, 5.2)	t091 (10, 4.7)	10	0	/
		t796 (1, 0.5)	1		
CC59	ST59 (9, 4.2)	t163 (6, 2.8)	5	**1**	IV
		t437 (3, 1.4)	3	0	/
	ST537 (1, 0.5)	t2755 (1, 0.5)	0	**1**	IV
CC1	ST1 (5, 2.4)	t127 (3, 1.4)	3	0	/
		t114 (2, 0.9)	2		
	ST1920 (3, 1.4)	t693 (3, 1.4)	3		
	ST81 (1, 0.5)	t5100 (1, 0.5)	1		
CC9	ST9 (10, 4.7)	t899 (10, 4.7)	1	**9**	XII
CC151	ST151 (5, 2.4)	t529 (3, 1.4)	3	0	/
		t528 (2, 0.9)	2	0	
CC5	ST6 (4, 1.9)	t2360 (2, 0.9)	2	0	/
		t701 (2, 0.9)	2	0	
	ST965 (2, 0.9)	t062 (2, 0.9)	0	**2**	IV
CC20	ST20 (3, 1.4)	t164 (1, 0.5)	1	0	/
		t1987 (1, 0.5)	1		
		t2094 (1, 0.5)	1		
CC25	ST25 (2, 0.9)	t280 (2, 0.9)	2	0	/
CC88	ST88 (1, 0.5)	t3155 (1, 0.5)	1	0	/
CC630	ST630 (1, 0.5)	t4549 (1, 0.5)	1	**1**	V
CC50	ST50 (1, 0.5)	t518 (1, 0.5)	1	0	/
Total	212		198	14	

*The bold values highlight the STs of MRSA strains*.

**Figure 1:**
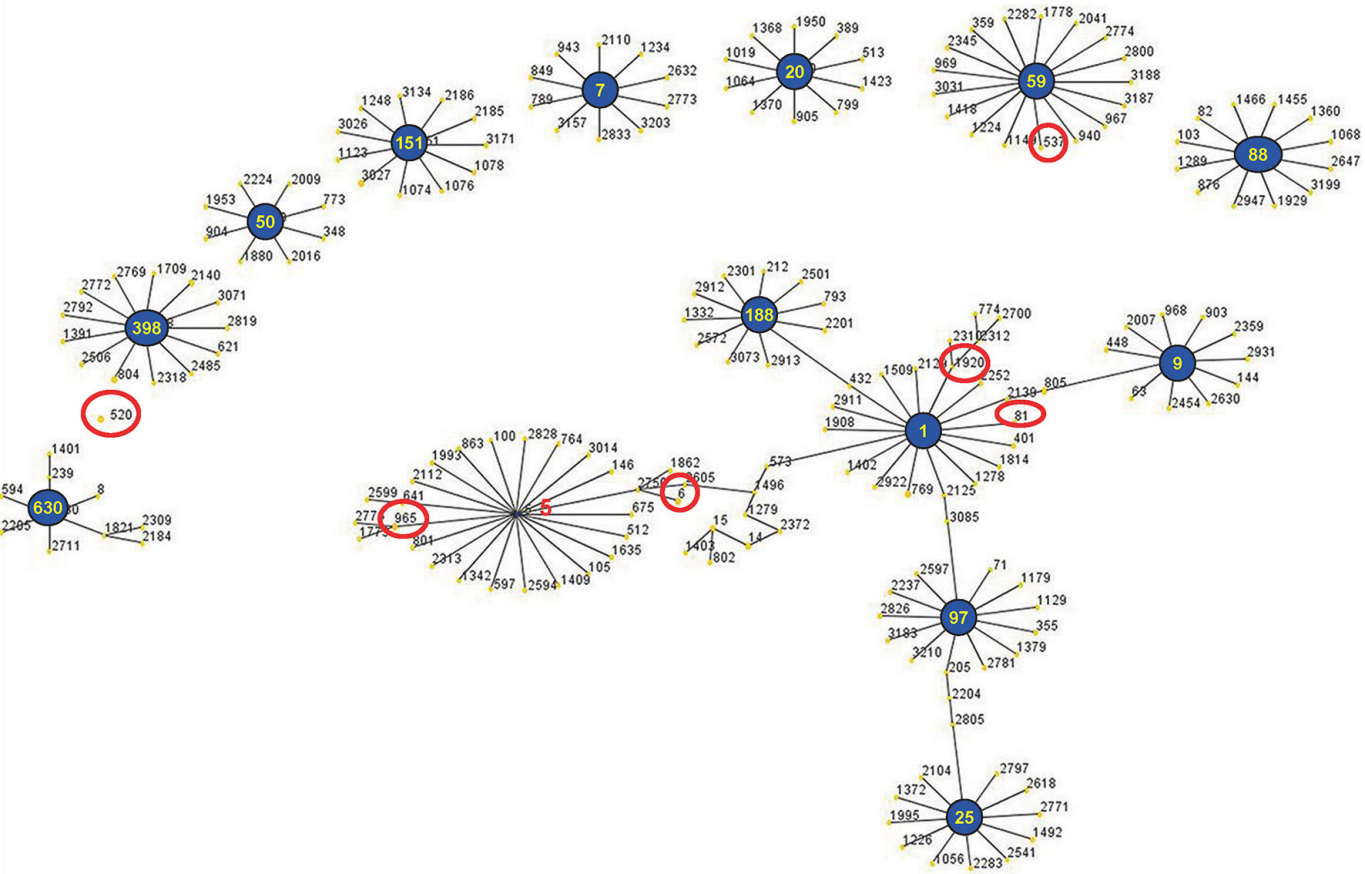
**Application of the eBURST algorithm to MLST data for the collection of 212 *S*. *aureus* isolates**. All 19 STs are represented by a filled circle. Blue and red circles represent STs that are group and sub-group founders, respectively. CC includes the groups of connected STs, considering that STs have at least six alleles in common with at least one other ST inside a CC.

The eBURST analysis of bovine-associated *S. aureus* using all STs available in the MLST database is shown. A population scatter diagram of the bovine-associated isolates shows nine main primary groups (Figure 1). The eBURST algorithm clustered all 19 STs isolated from bovine mastitis into 14 clonal complexes, as follows, CC1, CC9, CC97, CC25, CC188, CC630, CC398, CC50, CC151, CC7, CC20, CC59, CC88, and CC5. Among these complexes, CC97 had 83 isolates, CC520 had 34 isolates, CC188 had 21 isolates, CC398 had 15 isolates, CC7 had 11 isolates, CC59 had 10 isolates, and CC9 had 10 isolates. Each of other CCs had <10 isolates.

### Antimicrobial susceptibility profiles

The results of antibiotic susceptibility testing showed that all of the isolates were susceptible to vancomycin, teicoplanin, and linezolid. Although the majority of the strains were resistant to penicillin (73.1%), they were still susceptible to most of the antibiotics tested. The resistance rates to other antibiotics tested were 31.6% to gentamicin, 23.6% to erythromycin, 16.5% to clindamycin, 11.8% to ciprofloxacin, 6.6% to cefoxitin, or oxacillin, 4.7% to cefuroxime, 4.2% to sulfamethoxazole/trimethoprim, and 3.8% to cefazolin (Table 2).

**Table 2:** **Antimicrobial susceptibility profiles of *S. aureus* causing bovine mastitis in eastern regions of China arranged by STs**.

**Molecular type**	**Isolates (n)**	**CN^a^ (%)**	**KZ^a^ (%)**	**CXM^a^ (%)**	**OX^a^ (%)**	**SXT^a^ (%)**	**P^a^ (%)**	**DA^a^ (%)**	**E^a^ (%)**	**FOX^a^ (%)**	**CIP^a^ (%)**
ST97	83	45.8	0	0	0	0	90.4	6	15.7	0	1.2
ST520	34	2.9	0	0	0	2.9	2.9	2.9	2.9	0	0
ST188	21	19	0	0	0	0	76.2	9.5	23.8	0	19
ST398	15	40	0	0	0	0	86.7	53.3	93.3	0	66.7
ST7	11	9.1	0	0	0	0	100	9.1	18.2	0	9.1
ST9	10	100	80	80	90	80	100	90	40	90	80
Others (ST no < 10)	38	18.4	0	5.3	13.2	0	76.3	23.7	28.9	13.2	2.6
MRSA	14	64.3	57.1	71.4	100	50	100	78.6	64.3	100	50
MSSA	198	29.3	0	0	0	1.0	71.2	12.1	20.7	0	9.1
Total	212	31.6	3.8	4.7	6.6	4.2	73.1	16.5	23.6	6.6	11.8

a *CN, Gentamicin; KZ, Cefazolin; CXM, Cefuroxime; OX, Oxacillin; SXT, Sulfamethoxazole/trimethoprim; P, Penicillin; DA, Clindamycin; E, Erythromycin; FOX, Cefoxitin; CIP, Ciprofloxacin*.

Among the 212 *S. aureus* isolates, 50 (23.6%) strains were resistant to ≥3 antibiotics, including 14 (6.6%) MRSA and 36 (17.0%) MSSA strains. Nineteen (9.0%) isolates were resistant to five or more antibiotics, seventeen (8.0%) were resistant to four antibiotics and fourteen (6.6%) were resistant to three antibiotics. In the MSSA strains, 36 (17.0%) strains were resistant to ≥3 antibiotics, 22 (10.4%) strains showed resistance to ≥4 antibiotics, and 7 (3.3%) strains were resistant to ≥5 antibiotics. However, all the MRSA strains were found to be resistant to at least four antibiotics.

The resistance profiles of *S. aureus* isolates differed by their STs (Table 2). ST9 strains displayed the MDR phenotype, with high resistance (≥80%) to nine of the antibiotics tested except erythromycin (40%). ST398 isolates showed a higher resistance rate to erythromycin (93.3%), penicillin (86.7%), and ciprofloxacin (66.7%). ST97, ST188, and ST7 isolates were more resistant to penicillin (90.4, 76.2, and 100%, respectively) than to other antibiotics. Among 34 ST520 strains, only nine were resistant to one or two types of antibiotics, and the other ST520 isolates were susceptible to all antibiotics tested.

### Detection of virulence genes

In the present study, 30 virulence genes were screened in six predominant STs. The distribution of virulence genes differed among the strains according to ST. Some genes were present in all of the strains, but some genes were not found in any strain. For example, all of the strains carried four hemolysin genes (*hla, hlb, hld, hlg*), one serine protease gene (*sspA*), and three adhesion genes (*icaA, clfA, fnbA)*, but none of the strains harbored *tst, arcA, eta, etb*, or *pvl*. The carriage of staphylococcal enterotoxin genes was strongly influenced by MLST profiles. Thirteen staphylococcal enterotoxin genes (*sea, seb, sec, sed, see, seg, seh, sei, sem, sen, seo, seq, sek*) were detected among these strains. Some enterotoxin genes (*sea, seb, sed, seh, sej, sek, seq*) could not be found in any strain. No enterotoxin gene was found in ST97, ST188, or ST398 isolates. The *see-sep* genes were only present in the ST7 strain, but the *seg-sei-sem-sen-seo* genes were present in ST7, ST9, and ST520 strains. Compared to the other STs, ST520 had the highest carrying frequency. The *seg-sei-sem-sen-seo* genes were present in all 10 ST520 isolates (Table 3). Leucotoxins are regarded as virulence factors of *S. aureus*. Only ST520 (70%) carried both *lukE* and *lukM. LukM* could not be found in other STs, but *lukE* was detected in various other STs, including ST97, ST188, and ST7. The serine-aspartate repeat proteins (*sdr*) are members of a family of surface proteins that contribute to the pathogenicity of *S. aureus*. ST97 and ST9, which were the predominant STs of MSSA and MRSA, respectively, contained both *sdrD* and *sdrE*. Furthermore, *sdrD* only existed in ST7, but *sdrE* was present in various other STs, such as ST520, ST188, and ST398.

**Table 3:** **Analysis of virulence genes carrying of the main clones**.

**ST types**	**Virulence genes (%)**										
	***see***	***seg***	***sei***	***sem***	***sen***	***seo***	***sep***	***lukE***	***lukM***	***sdrD***	***sdrE***
ST97	0	0	0	0	0	0	0	100	0	100	100
ST520	0	100	100	100	100	100	0	90	70	0	100
ST188	0	0	0	0	0	0	0	100	0	0	80
ST398	0	0	0	0	0	0	0	0	0	0	100
ST7	40	10	10	10	10	10	40	100	0	100	0
ST9	0	20	20	20	20	20	0	0	0	100	100

*STs with <10 isolates were not included when calculating the percentages of virulence genes*.

### Biofilm formation and human erythrocyte lysis in *S. aureus* strains

**Figure 2:**
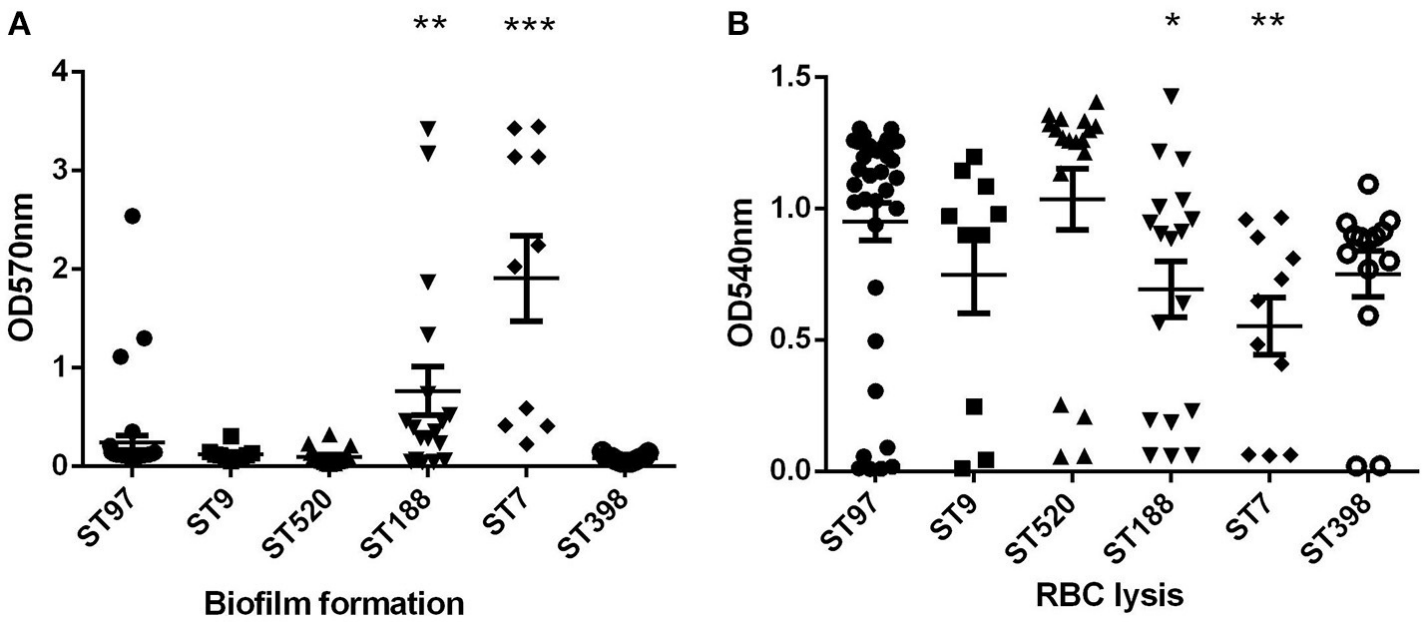
**Biofilm formation and red blood cells lysis. (A)** Semi-quantitative biofilm analysis of STs with more than 10 isolates. ^***^P < 0.001 (unpaired *t*-test, ST7 vs. ST97); ^**^P < 0.01 (unpaired *t*-test, ST188 vs. ST97). **(B)** Hemolytic capacities of STs with more than 10 isolates. ^*^P < 0.05 (unpaired *t*-test, ST188 vs. ST97) ^**^P < 0.01 (unpaired *t*-test, ST7 vs. ST97).

The ability of *S. aureus* to form biofilms is considered an important virulence factor because biofilms can tolerate antimicrobial agents, making the bacterium extremely difficult to eradicate. The biofilm assay was performed in the ST97 (randomly selected, *n* = 39), ST520 (randomly selected, *n* = 18), ST188 (randomly selected, *n* = 10), ST398 (randomly selected, *n* = 10), ST7 (randomly selected, *n* = 10), and ST9 (*n* = 10) strains. Compared to ST97 isolates, biofilm formation ability was enhanced significantly in ST188 and ST7 strains (Figure 2A). Moreover, ST7 strains had stronger biofilm formation than ST188 isolates (*P* < 0.05). There were no significant differences among the other STs, including ST97, ST9, ST520, and ST398.

Hemolysis (lysis of erythrocytes) is also a significant virulence determinant of *S. aureus*, representing a crucial means for the bacteria to acquire iron. Lysis of red blood cell capacity was relatively strong in all *S. aureus* strains of animal origin, except ST7 (Figure 2B). The hemolytic capacities of ST7 and ST188 strains were weaker than in ST97 isolates. Among these strains, ST7 had the strongest biofilm and the hemolytic capacity.

## Discussion

*S. aureus* is the most common causative organism of bovine mastitis, which is considered a common, complicated and economically unbearable disease in dairy animals worldwide (Wyder et al., 2011). Dairy animals with mastitis frequently shed *S. aureus* into the milk supply, which can contribute to food poisoning in humans. This study intended to investigate the molecular profile and antimicrobial resistance of *S. aureus* isolates recovered from bovine mastitis from 2014 to 2015 in eastern regions of China.

In this study, we characterized 212 *S. aureus* isolates from bovine mastitis milk samples collected from farms in Shanghai and Zhejiang areas and identified 6.6% as MRSA strains, which was lower than western countries and Hong Kong (16–21.3%; Guardabassi et al., 2009), but higher than Korea (21/657, 3.2%; Lim et al., 2012), Malaysia (1.4%; Neela et al., 2009), and Japan (0.9%; Baba et al., 2010). These different frequencies may be due to the different animal populations studied or the implemented methodologies, among other factors.

Our study identified 14 bovine MRSA strains, including nine ST9-SCC*mec*XII-*t899* strains, *four* belonging to SCC*mec* IV type (two ST965-*t062*, one ST59-*t437*, one ST537-*t2755*), and one SCC*mec*V strain (ST630-*t4549*). The MRSA isolates from eastern regions of China belonged to different *spa* and SCC*mec* types, suggesting their genetic diversity. In those previous studies, CC9 was identified as the predominant MRSA associated with pig farming and harbored various SCC*mec* types in different areas. For example, SCC*mec* was type III in mainland China, types IVb or V in Hong Kong, non-types I–VII in Taiwan, type V in Malaysia and type IX in Thailand (Cui et al., 2009; Ho J. et al., 2012; Larsen et al., 2012). In addition, SCC*mec* XII was a new SCC*mec* element that was first identified in two ST9-MRSA-*t899* strains from pigs in China (Ho P. L. et al., 2012; Wang et al., 2015; Wu et al., 2015). It included a rare class C2 *mec* gene complex and a *ccrC* gene complex. The newly identified SCC*mec* was 48.575 kb in length and contained 45 predicted open reading frames. Therefore, the emerging ST9-MRSA-SCC*mec* XII strains can be isolated in both pigs and bovines in China, suggesting the potential possibility of its cross-species transmission. Notably, the CC9 MRSA isolates in the current study were shown to be multidrug-resistant, with high resistance rates to β-lactams and eight other antibiotics widely used in chemotherapy. The presence of multidrug resistant MRSA clone CC9 suggests an alarming situation and could be a serious challenge to therapy. The PFGE result in our study showed that the restriction profiles of 10 CC9 strains were identical with only one fragment difference between MRSA CC9 and MSSA CC9 (Supplementary Image 1). These observations support the notion that CC9 MSSA lineages may provide a stable genetic environment for the integration of SCC*mec* in favor of their infection and transmission in different areas.

Among the 212 *S. aureus* isolates, the prevalence of MSSA in this study was 93.4%. In addition, our study revealed more heterogeneous MSSA lineages, 16 distinct STs were identified in MSSA, but only five STs were found in MRSA. The most frequently represented lineage in MSSA was CC97 (39.2%, 83/212), which accounted for more than one third of all *S. aureus* isolates, followed by CC520 (16%, 34/212), CC188 (10%, 21/212), CC398 (7.1%, 15/212), and CC7 (5.2%, 11/212). CC97 was found to be the most predominant clone, which is consistent with a recent study on bovine mastitis infection that also identified CC97 as the dominant strain type among six provinces of China from March 2010 to August 2013 (Wang et al., 2015). However, the difference is that all CC97 isolates identified in our study were MSSA strains. In particular, CC97 strains have become the dominant lineage in Chile (Smith et al., 2005), Brazil (Aires-de-Sousa et al., 2007; Rabello et al., 2007), Japan (Hata et al., 2010), the Netherlands (Ikawaty et al., 2009), and the United States (Smith et al., 2005). We found that CC97 isolates were more resistant to gentamicin and penicillin than other MSSA isolates. This implies that appropriate drug selection based on different MSSA types may reduce the reservoir of drug-resistant bacteria.

The second most frequent MLST type shown here was CC520 (16%), which was only reported in a few studies and could indicate the emergence of a new epidemic clone. Compared to other lineages, CC520 carried more virulence elements but presented a lower drug resistance rate, which may facilitate its success as a pathogen spread in bovines. Moreover, in our study, 21 MSSA isolates belonged to CC188, which is a double locus variant (DLV) of CC1 that includes MW2/USA400, the highly virulent and first-known Panton-Valentine leukocidin (PVL)-positive MRSA strain (Centers for Disease Control and Prevention, 1999). In a previous study of human infection, CC188 was the predominant clone in community-onset MSSA infections (Chen et al., 2012). However, the contamination of bovine mastitis samples with *S. aureus* by humans cannot be excluded. We propose that these results represent a probable transient acquisition from human handling, which will require more attention in further studies.

LA MRSA was originally reported to have emerged in 2003 among healthy pigs and pig farmers in France and the Netherlands (Witte et al., 2007). In European countries and in North America, CC398 was the overwhelmingly dominant LA MRSA, whereas CC9 predominated in most Asian countries (Cortimiglia et al., 2016; Sharma et al., 2016). In our study, all CC398 strains were identified as MSSA strains. Additionally, the observed *spa* type distribution of MSSA CC398 mirrored the most frequently detected *spa* types for MRSA CC398: *t034*. In contrast to the large number of reports on MRSA CC398 (Fessler et al., 2010; Schijffelen et al., 2010; Smith and Pearson, 2010; Crombé et al., 2012; Price et al., 2012), limited attention has been given to the ecology of MSSA CC398 in livestock farms (Hasman et al., 2010). MSSA CC398 is a clone of clinical importance that affects humans and livestock in different geographic regions. Price et al. analyzed 89 isolates and concluded that LA-MRSA likely originates from humans as MSSA. They theorized that the recent emergence of LA-MRSA can be seen as a reintroduction of MSSA that acquired methicillin-resistance to the original host (Price et al., 2012). Therefore, CC398-MSSA strains in this study may be a risk factor for the transmission of MRSA to people in close contact with the infected animals. Additionally, the present study showed that CC398 was more resistant to clindamycin, erythromycin, and ciprofloxacin. Our results may contribute greatly to the understanding of the emergence and transmission of MRSA CC398 among livestock because they may indicate the existence of a reservoir from which MRSA CC398 strains can emerge.

*S. aureus* is the most common causative organism of bovine mastitis. However, the treatment and control of this infection often fails due to the complex nature of the *S. aureus* strains. The development of drug resistance is a significant feature of these organisms. The results of this research revealed that all MRSA isolates were resistant to oxacillin, penicillin and cefoxitin. All MSSA isolates were susceptible to cefazolin, cefuroxime, and oxacillin. In general, MRSA strains showed much higher resistance rates to all the tested antibiotics than MSSA strains (Table 3). All the MRSA strains were found to be resistant to at least four antibiotics. It was apparent that CC9 strains had significantly higher multiple antibiotic resistance profiles compared with other lineages. The widespread resistance of cattle-derived *S. aureus* will be a serious challenge to bovine mastitis therapy. The ability of bacterial pathogens to produce biofilms is regarded as a major cause of resistance to antibiotics and as responsible for persistent infections and innate immune defense for both animal and human infectious diseases (Cucarella et al., 2004; Bjarnsholt, 2013). The biofilm formed in multilayer growth of bacteria, avoiding bacterial clearance by the host immune system. In our study, we detected biofilm formation in different lineages. The results showed that the biofilm formation ability of CC7 and CC188 strains was significantly higher than other lineages. However, the resistance rates to different antibiotics in CC7 and CC188 were not higher than other lineages. This may indicate that the mechanism of drug resistance in bovine mastitis-associated *S. aureus* may depend on other factors than biofilm formation. While biofilm formation increases drug resistance and infection time, it can reduce bacterial virulence. Hemolysis (lysis of erythrocytes) is a significant virulence determinant of *S. aureus*, representing a crucial way for the bacteria to acquire iron. As shown in our study, lysis of red blood cell capacity was relatively strong in all *S. aureus* strains of animal origin, except CC7 and CC188. Therefore, different types of *S. aureus* have different potential risks to human beings.

In conclusion, the prevailing *S. aureus* strains in bovine mastitis were mainly MSSA strains in eastern regions of China. However, the presence of the multidrug-resistant and toxigenic MRSA clone ST9-t899 SCC*mec* XII suggests an alarming situation and might be a serious challenge to therapy, creating a public health concern. There were differences in the resistance genes, virulence genes and biological activities of the different molecular types of strains. As *S. aureus* from livestock may emerge as a threat to public health, comprehensive surveillance should be taken against *S. aureus* infection in the management of animal husbandry products.

## Ethics statement

Blood of healthy individuals for the lysis of erythrocytes by bacterial culture was collected using a standard method in accordance with a protocol approved by the ethics committee of Renji Hospital, School of Medicine, Shanghai Jiaotong University, Shanghai, China. All individuals provided written informed consent prior to donating blood.

## Author contributions

ML, JS, and XW planned and supervised the experiments and wrote the paper. TL, HL, XW, QG, and YD performed the experiments and/or analyzed the data.

## Funding

This work was supported by the National Natural Science Foundation of China (grants 81371875, 81671975, and 81601737), the Shanghai Committee of Science and Technology, China (grant 15411960500) and the Foundation for Innovative Research Groups of the National Natural Science Foundation of China (grant 81421001).

### Conflict of interest statement

The authors declare that the research was conducted in the absence of any commercial or financial relationships that could be construed as a potential conflict of interest.

## Supplementary material

Supplementary Image 1 **Pulsed-field gel electrophoresis (PFGE) of SmaI macro-restriction fragments of 10 bovine mastitis-associated isolates of ST9 isolated in this study**. Lane M, XbaI-digested DNA of *Salmonella enterica* serovar as a reference molecular size marker. Lanes 1–10: 10 isolates of ST9 (Lane 1–5, 7–10 were MRSA ST9, Lane 6 was MSSA ST9).
