# Supplementary figures and images for: Molecular Characteristics of Staphylococcus aureus Causing Bovine Mastitis between 2014 and 2015
Source: Front Cell Infect Microbiol. 2017 Apr 19;7:127. doi: 10.3389/fcimb.2017.00127 (PMC5395632; doi:10.3389/fcimb.2017.00127)

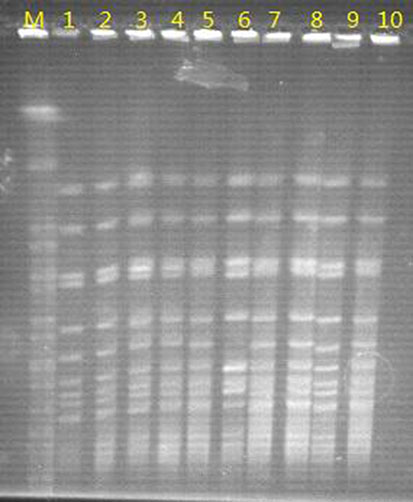

Supplement: Supplementary Image 1 — Pulsed-field gel electrophoresis (PFGE) of SmaI macro-restriction fragments of 10 bovine mastitis-associated isolates of ST9 isolated in this study. Lane M, XbaI-digested DNA of Salmonella enterica serovar as a reference molecular size marker. Lanes 1–10: 10 isolates of ST9 (Lane 1–5, 7–10 were MRSA ST9, Lane 6 was MSSA ST9). [file Image1.JPEG]
